# DeSUMOylation of Apoptosis Inhibitor 5 by *Avibirnavirus* VP3 Supports Virus Replication

**DOI:** 10.1128/mBio.01985-21

**Published:** 2021-08-10

**Authors:** Tingjuan Deng, Boli Hu, Xingbo Wang, Yan Yan, Jianwei Zhou, Lulu Lin, Yuting Xu, Xiaojuan Zheng, Jiyong Zhou

**Affiliations:** a MOA Key Laboratory of Animal Virology, Zhejiang University Center for Veterinary Sciences, Hangzhou, Zhejiang, People’s Republic of China; b State Key Laboratory for Diagnosis and Treatment of Infectious Diseases, First Affiliated Hospital, Zhejiang University, Hangzhou, Zhejiang, People’s Republic of China; Virginia Polytechnic Institute and State University

**Keywords:** *Avibirnavirus* VP3, apoptosis inhibitor 5, SUMOylation, virus replication, IFN-β

## Abstract

SUMOylation is a reversible posttranslational modification involved in the regulation of diverse biological processes. Growing evidence suggests that virus infection can interfere with the SUMOylation system. In the present study, we discovered that apoptosis inhibitor 5 (API5) is a SUMOylated protein. Amino acid substitution further identified that Lys404 of API5 was the critical residue for SUMO3 conjugation. Moreover, we found that *Avibirnavirus* infectious bursal disease virus (IBDV) infection significantly decreased SUMOylation of API5. In addition, our results further revealed that viral protein VP3 inhibited the SUMOylation of API5 by targeting API5 and promoting UBC9 proteasome-dependent degradation through binding to the ubiquitin E3 ligase TRAF3. Furthermore, we revealed that wild-type but not K404R mutant API5 inhibited IBDV replication by enhancing MDA5-dependent IFN-β production. Taken together, our data demonstrate that API5 is a UBC9-dependent SUMOylated protein and deSUMOylation of API5 by viral protein VP3 aids in viral replication.

## INTRODUCTION

SUMOylation, an important reversible posttranslational modification (PTM), occurs widely and has been implicated in numerous key cellular functions through regulating subcellular localization, signaling pathways, or the activity of target proteins (1–4). The small ubiquitin-like modifier SUMO can be conjugated to lysine (K) residues of substrate proteins via an enzymatic cascade involving E1-activating enzyme (SUMO-activating enzymes 1 and 2 [SAE1/2]), E2-conjugating enzyme (UBC9), and E3 SUMO ligases (5, 6). Four different SUMO isoforms have been detected in mammalian cells, including SUMO1, the highly related proteins SUMO2 and SUMO3, and SUMO4. SUMO2 and SUMO3 have 97% sequence identity, such that antibodies cannot distinguish them; therefore, SUMO2 and SUMO3 are often referred to as SUMO2/3 (7, 8). *In vivo*, SUMO2/3 can form poly-SUMO chains, which are then conjugated to target proteins; however, SUMO1 does not have this feature (9–12). Thus, SUMO1 and SUMO2/3 may serve distinct functions and covalently attach to different substrate proteins *in vivo*. UBC9, a unique SUMO-conjugating enzyme, is the key protein of the SUMO machinery and functions as a hub for protein SUMOylation and transfers SUMO to targets (13). Recently, growing evidence indicates that viruses can interfere with the SUMOylation of proteins (14–16). In particular, UBC9 is frequently targeted by numerous viral proteins to create a conducive environment for their propagation, i.e., human papillomavirus E6/E7 oncoprotein (17), avian adenovirus early protein Gam1 (18), and human adenovirus oncoprotein E1A (19).

Apoptosis inhibitor 5 (API5), also known as antiapoptosis clone 11, was first reported to support cell viability under serum and growth factor deprivation conditions, potentially by inhibiting caspase-3 or E2F transcription factor 1 (E2F1)-induced apoptosis (20–22). It was then observed that API5 is overexpressed by many types of tumors to promote their progression and serves as a marker of poor prognosis for these cancers (23–28). Recently, API5 was reported to induce dendritic cell and immune response activation via Toll like receptor 4 (TLR4) and to target the nucleoprotein (NP) of influenza A virus to regulate virus replication (29, 30). Study in our laboratory indicated that API5 is upregulated in *Avibirnavirus*-infected DF-1 cells and translocates to the cytoplasm from the nucleus (31). However, the role of API5 in *Avibirnavirus* infection is poorly understood.

*Avibirnavirus* infectious bursal disease virus (IBDV), a member of the *Birnaviridae* family, is a nonenveloped RNA virus containing a bi-segmented double-stranded RNA (dsRNA) genome comprising segment A and segment B (32). Segment B encodes the putative RNA-dependent RNA polymerase VP1 (33). Segment A encodes nonstructural protein VP5 and the precursor polyprotein NH3-pVP2-VP4-VP3-COOH, which can be self-cleaved to form pVP2, VP3, and VP4 (34). Recently, we found that *Avibirnavirus* VP1 can employ the host posttranslational modification system to support viral replication (35, 36). In addition, reports demonstrated that VP3, a scaffolding protein with multiple functions, can inhibit the phosphorylation of dsRNA-dependent protein kinase R (PKR) to promote IBDV replication (37). However, whether viral protein VP3 can affect viral replication by modulating small ubiquitin-like modification of cellular proteins is unknown.

The objective of this study was to explore whether API5 undergoes SUMOylation and, if so, how to regulate its function and whether *Avibirnavirus* VP3 can regulate self-replication through interfering with API5 SUMOylation. Here, we revealed that API5 K404 can be conjugated by SUMO3. Meanwhile, we demonstrated that *Avibirnavirus* infection inhibited API5 SUMOylation. Further investigation of the molecular mechanism demonstrated that VP3 directly inhibited SUMO3 modification of API5 via their interaction and facilitated UBC9 degradation. Finally, we revealed that the deSUMOylation of API5 at K404 supports IBDV replication by blocking MDA5-dependent IFN-β induction.

## RESULTS

### API5 is a SUMOylated protein.

**FIG 1:**
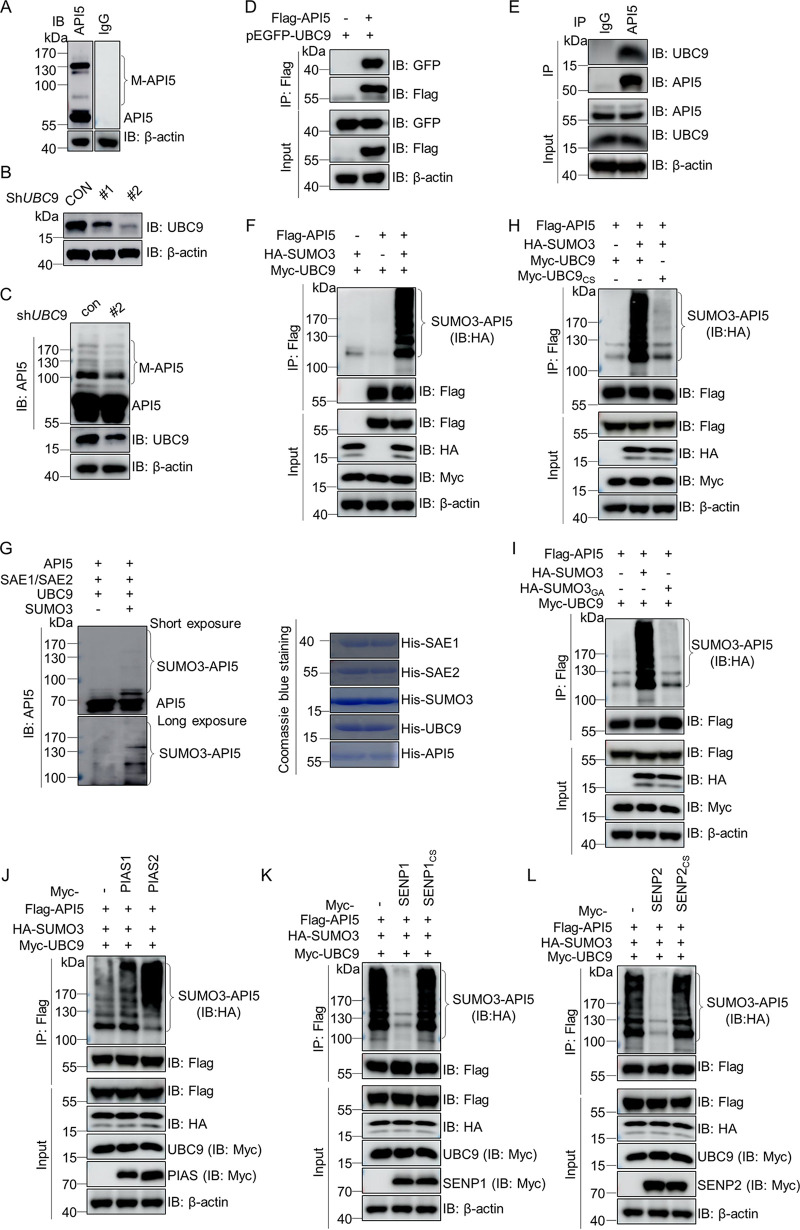
API5 can be conjugated by SUMO3. (A) Modified bands of endogenous API5. Lysates from DF-1 cells were analyzed using Western blotting with anti-IgG and anti-API5mouse MAbs. (B) Selecting an effective shRNA against *UBC9*. DF-1 cells were transfected with shRNA against *UBC9*. One control shRNA (shCON) and two *UBC9* shRNA (#1, #2) transfected DF-1 cells were subjected to Western blotting using anti-UBC9 antibodies; β-actin expression served as a loading control. (C) Decrease in SUMOylated API5 in *UBC9* knockdown DF-1 cells. DF-1 cells were transfected with shCON and *UBC9* shRNA (shUbc9#2) for 48 h and then analyzed using Western blotting with anti-API5 mouse MAb. (D) API5 interacts with UBC9 during transfection. HEK293T cells were cotransfected with Flag-API5 and pEGFP-UBC9 for 48 h and then subjected to a co-IP assay with anti-Flag mouse MAb and Western blotting with anti-Flag and anti-GFP rabbit pAbs. (E) Endogenous API5 associates with UBC9 in DF-1 cells. The lysates from DF-1 cells were immunoprecipitated with anti-IgG or anti-API5 mouse MAbs and immunoblotted using anti-API5 and anti-UBC9 rabbit pAbs. (F) Efficient modification of API5 by SUMO3. Flag-API5, HA-SUMO3, and Myc-UBC9 were transfected into HEK293T cells for 48 h and then subjected to a SUMOylation assay with anti-Flag mouse MAb and Western blotting with anti-HA mouse MAb, anti-Flag, and anti-Myc rabbit pAbs. (G) API5 can be conjugated by SUMO3 *in vitro*. IPTG-inducible His-tagged recombinant proteins E1 activating enzyme (SAE1/SAE2), E2 conjugated enzyme (UBC9), SUMO3, and API5 were expressed in E. coli BL21 and purified using Ni^2+^ column affinity pulldown. These purified recombinant proteins were identified by Coomassie blue staining (right). Recombinant API5 was used as a substrate for an *in vitro* SUMOylation assay in the presence of SUMO3 and UBC9. The reaction products were analyzed using Western blotting with anti-API5 mouse MAb (left). (H and I) The active sites of UBC9 and SUMO3 are necessary for API5 SUMOylation. HEK293T cells were cotransfected with Flag-API5, HA-SUMO3, Myc-UBC9, and Myc-UBC9_C93S_ or HA-SUMO3_G92A_ for 48 h and then subjected to a SUMOylation assay using anti-Flag mouse MAb and Western blotting with anti-HA mouse MAb and anti-Flag and anti-Myc rabbit pAbs. (J to L) PIAS2, SENP1, and SENP2 participate in API5 SUMOylation. Flag-API5, HA-SUMO3, Myc-UBC9, and Myc-PIAS1 or Myc-PIAS2, Myc-SENP1 or Myc-SENP1_C603S_, Myc-SENP2 or Myc-SENP2_C548S_ were cotransfected into HEK293T cells for 48 h. The lysates were subjected to a SUMOylation assay using anti-Flag mouse MAb and Western blotting with anti-HA mouse MAb and anti-Flag and anti-Myc rabbit pAbs.

To explore the chemical modification of API5, lysates from DF-1 cells were analyzed. In immunoblotting assays, we observed the expected band with an approximate molecular weight of 55 kDa (API5) and also higher molecular weight bands (termed M-API5) (Fig. 1A). To determine whether the posttranslational modification of API5 involves SUMOylation, we screened highly efficient short hairpin RNA (shRNA) against *UBC9* in DF-1 cells (Fig. 1B) and observed that M-API5 levels were significantly reduced in *UBC9*-silenced DF-1 cells compared with control cells (Fig. 1C). In addition, Fig. 1D and E show that both exogenous and endogenous API5 interacted with UBC9. These data indicated that M-API5 might represent SUMOylated API5. Meanwhile, *in vivo* and *in vitro* SUMOylation assays indicated that SUMO3-conjugated API5 could be detected (Fig. 1F and G), suggesting that API5 can be effectively modified by SUMO3.

Residue 93 (cysteine [C]) of UBC9 and residue 92 (glycine [G]) of SUMO3 were reported to be the active sites for the SUMOylation of substrates (38). We constructed Myc-Ubc9_C93S_ and HA-SUMO3_G92A_ mutants. SUMOylation assays revealed that SUMO3-API5 levels were reduced significantly in Ubc9_C93S_- and SUMO3_G97A_- transfected cells (Fig. 1H and I), indicating that SUMOylation of API5 is UBC9- and SUMO3-dependent. SUMOylation of substrates is commonly controlled by SUMO E3 ligases, such as protein inhibitor of activated STAT (PIAS), and deSUMOylation enzymes (sentrin-specific proteases, SENPs) (39, 40). To determine the SUMO3 E3 ligases and deSUMOylateases of API5, we constructed multiple PIAS and SENP expression plasmids. Finally, the results showed that PIAS2 increased SUMO3 modification of API5 (Fig. 1J), and SENP1 and SENP2 exhibited the most efficient deconjugation effect (Fig. 1K and L). Amino acids C603 of SENP1 and C548 of SENP2 function as the active sites of SUMO covalent attachment (41, 42). We constructed SENP1_CS_ and SENP2_CS_ mutants, and further confirmed that SENP1 and SENP2 were responsible for reversing API5 SUMO3 modification (Fig.1K and L). Taken together, these data indicated that SUMO3 modification of API5 is regulated by PIAS2, SENP1, and SENP2.

### Lysine 404 of API5 is the SUMO3 modification site.

**FIG 2:**
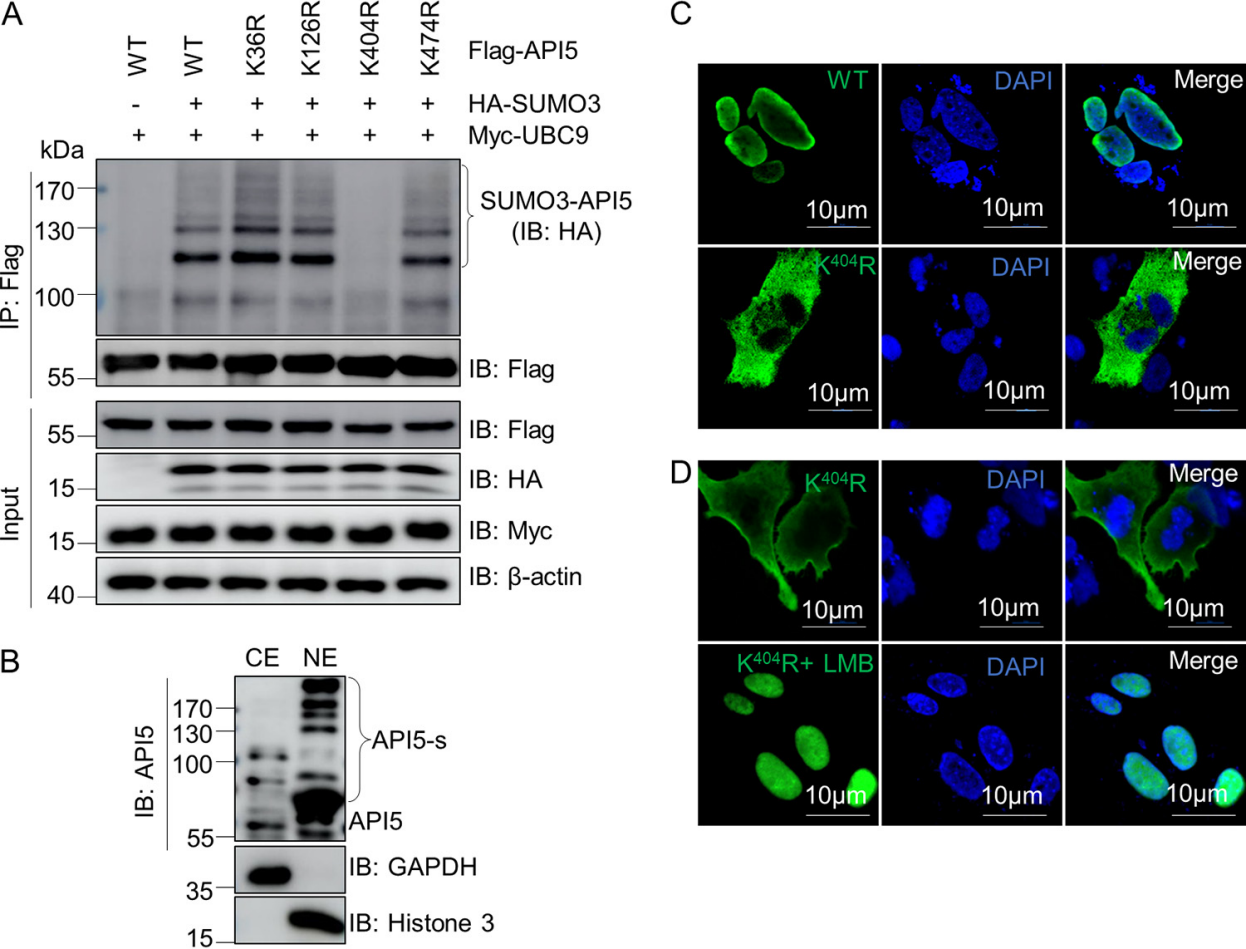
K404 of API5 is required for its SUMO3 modification. (A) K404 of API5 was identified as the SUMOylation site. HEK293T cells were cotransfected separately with HA-SUMO3, Myc-UBC9, and Flag-API5 or its various mutants, for 48 h. Lysates from the resulting cells were subjected to a SUMOylation assay with anti-Flag mouse MAb and to Western blotting using anti-HA mouse MAb and anti-Flag and anti-Myc rabbit pAbs. (B) SUMOylated API5 mainly localizes to the nuclei. Cytoplasmic and nuclear components from DF-1 cells were analyzed using Western blotting with anti-API5 mouse MAb. GAPDH and histone 3 were used as markers of the cytosolic and nuclear fractions, respectively. (C) DF-1 cells were transfected with Flag-API5-WT or Flag-API5K^404^R mutant for 48 h. (D) Flag-API5K^404^R was transfected into DF-1 cells for 36 h and the resulting cells were treated with leptomycin B (20 nM) for 12 h. The cells in C and D were subjected to immunofluorescence staining assays with anti-Flag mouse MAb primary antibody and FITC-labeled goat anti-mouse secondary antibody. DAPI staining revealed the nuclei. Finally, the cells were analyzed by confocal microscopy. Scale bar, 10 μm.

To determine the SUMO3 modification site of API5, we generated a series of API5 mutants according to the results of online SUMOylation prediction (http://sumosp.biocuckoo.org/). Figure 2A shows that SUMO3 greatly increased the SUMOylation of API5-WT (wild type), K36R, K126R, and K474R mutants, but not the K404R mutant, suggesting that the residue K404 is critical for the SUMO3 modification of API5. To explore if SUMO modification of API5 (API5-s) affected its nuclear transport, cytoplasmic and nuclear components from DF-1 cells were analyzed. Immunoblotting results showed that API5-s was mainly located in the nucleus (Fig. 2B). Moreover, confocal microscopy revealed that API5 K404R failed to localize to the nucleus (Fig. 2C) and this mutant localized in the nucleus in cells treated with leptomycin B, an inhibitor of nuclear export (Fig. 2D), indicating that SUMO3 modification at K404 contributes to the nuclear retention of API5. Collectively, these results demonstrated that K404 of API5 is important for its SUMO3 modification and nuclear localization.

### Residue L211 on the CC3 domain of VP3 is critical for direct interaction with API5.

**FIG 3:**
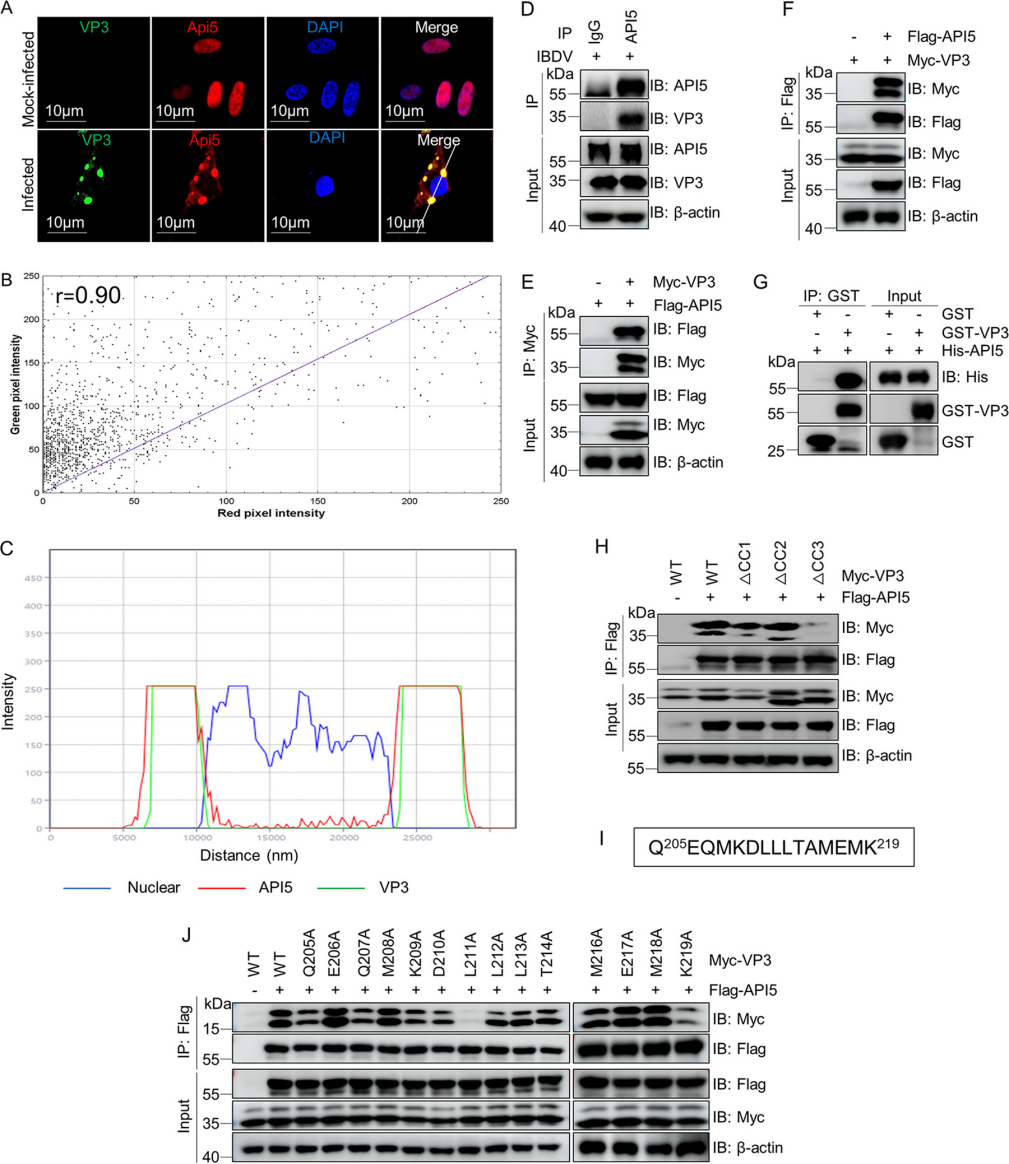
The CC3 domain of VP3 participates in the direct interaction with API5. (A) VP3 colocalizes with API5 in cytoplasm with IBDV. DF-1 cells were transfected with Flag-API5 for 24 h and then infected for another 12 h with IBDV. Cells were stained with anti-VP3 mouse MAb and anti-Flag rabbit pAb, followed by FITC-labeled goat anti-mouse IgG and Alexa Fluor 546-conjugated donkey anti-rabbit IgG antibodies, and finally with DAPI, before being observed by confocal microscopy. Scale bar, 10 μm. (B) Scatterplot. The Image J Just Another Colocalization Plugin (JACoP) was applied for quantitative colocalization analysis. In the scatterplot, the symbol *r* represents the Pearson's correlation coefficient. (C) Relative intensity of the three channels along the white line in A. (D) Viral protein VP3 interacts with endogenous API5. DF-1 cells were infected for 12 h with IBDV. The resulting cells were lysed for co-IP assays using mouse MAb against API5. The interaction between VP3 and API5 was detected using Western blotting with anti-VP3 mouse MAb and anti-API5 rabbit pAb. (E and F) VP3 associates with API5 during transfection. Flag-API5 and Myc-VP3 were cotransfected into HEK293T cells for 48 h and then cellular lysates were separately subjected to co-IP with anti-Flag or anti-Myc mouse MAb, followed by Western blotting with anti-Flag and anti-Myc rabbit pAbs. (G) VP3 directly targets API5 *in vitro*. Equal amounts of purified GST and GST-VP3 proteins were separately incubated with His-API5 for 4 h at 4°C. GST pulldown assays were performed. (H) The CC3 domain of VP3 is necessary for interaction with API5. Flag-API5 and Myc-VP3 or different truncated mutants (Myc-VP3ΔCC1, Myc-VP3ΔCC2, and Myc-VP3ΔCC3) were cotransfected separately into HEK293T cells for 48 h. Lysates were subjected to co-IP with anti-Flag mouse MAb and to Western blotting with anti-Flag and anti-Myc rabbit pAbs. (I) The sequence of residues of the CC3 domain on viral protein VP3. (J) The residue leucine (L)-211 of VP3 is crucial for the interaction with API5. Flag-API5 and Myc-VP3 or different mutants bearing substitution with alanine (A) were cotransfected into HEK293T cells for 48 h. Cellular lysates were subjected to co-IP assay using anti-Flag mouse MAb and immunoblotting assay with anti-Flag and anti-Myc rabbit pAbs.

*Avibirnavirus* VP3 protein is involved in interactions with cellular proteins and the modulation of numerous key cellular pathways (37, 43–45). Previous study in our laboratory showed that endogenous API5 colocalizes with VP3 in cytoplasm during infection (31). In the present study, we observed that overexpressed API5 colocalized with VP3 in the cytoplasm of IBDV-infected cells (Fig. 3A to C). To further explore the relationship between VP3 and API5, coimmunoprecipitation (co-IP) assays were conducted separately during infection and transfection. We found that VP3 interacted with API5 in IBDV-infected cells (Fig. 3D) and in cotransfected cells (Fig. 3E and F). In addition, glutathione *S*-transferase (GST) pulldown assays further confirmed that API5 interacted directly with VP3 (Fig. 3G). VP3 contains three coiled-coil (CC) domains, CC1, CC2, and CC3 (43). In co-IP assays, we further found that the residues 205 to 219 of the CC3 domain of VP3, but not CC1 and CC2, were required for the interaction with API5 (Fig. 3H and I). Substitution mutation assays further showed that the residue leucine (L) 211 on VP3 was essential for the association with API5 (Fig. 3J). These data revealed that the residue L211 on the CC3 domain of VP3 was responsible for binding to API5.

### VP3 inhibits SUMOylation of API5 via its CC3 domain with function similar to deSUMOylation enzyme.

**FIG 4:**
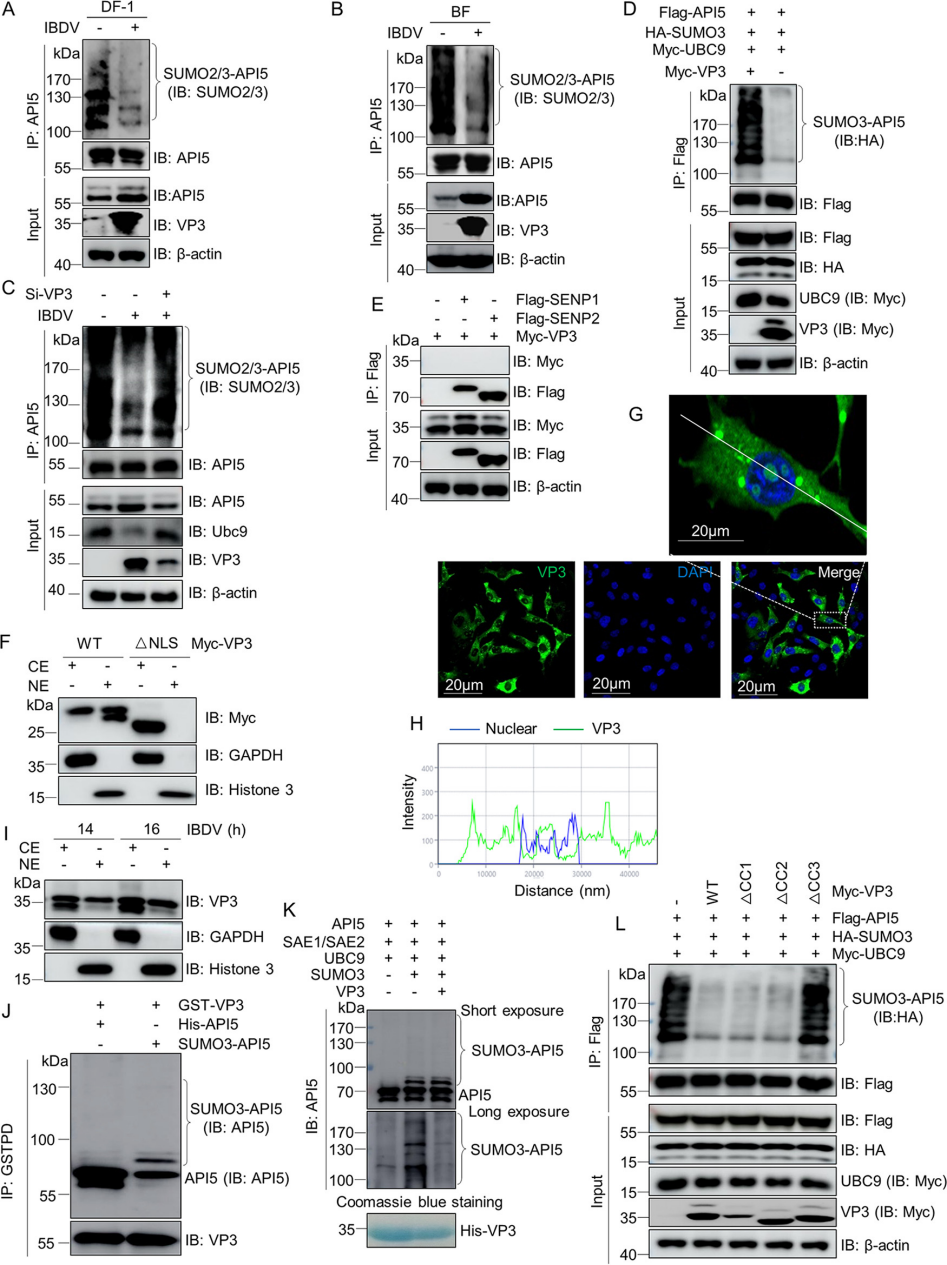
SUMOylation of API5 is decreased by the CC3 domain on VP3 with a function similar to that of the deSUMOylation enzyme. (A and B) SUMOylation of API5 was reduced during IBDV infection. Lysates from DF-1 cells infected with IBDV for 12 h (A) and bursae of fabricius (BF, IBDV-infected target organ) of 5-week-old specific-pathogen-free chickens infected with virulent IBDV strain NB at a dose of 0.1 ml (2 × 10^3^ 50% bird lethal doses/ml) for 72 h (B) were subjected to SUMOylation assays using the anti-API5 mouse MAb and Western blotting with anti-VP3 mouse MAb, anti-SUMO2/3, and anti-API5 rabbit pAbs. (C) Viral protein VP3 knockdown suppresses the reduction of SUMO2/3-API5 induced by IBDV. DF-1 cells were infected with IBDV at a multiplicity of infection (MOI) of 1. At 1 h after infection, the cells were transfected with the RNAi (GACGAAGUUGCCAAAGUCUAU) against VP3 for another 20 h using JetPRIME transfection reagent. Cellular lysates were subjected to the SUMOylation assay with anti-API5 mouse MAb and Western blotting using the indicated antibodies. (D) Viral protein VP3 inhibits SUMO3 modification of Api5. Flag-API5, HA-SUMO3, Myc-UBC9, and Myc-VP3 or empty vector were cotransfected into HEK293T cells for 48 h and then the cells were subjected to a SUMOylation assay using anti-Flag mouse MAb and Western blotting using anti-HA mouse MAb, anti-Myc, and anti-Flag antibodies. (E) VP3 cannot precipitate with SENP1 and SENP2. Flag-SENP1 or Flag-SENP2 and Myc-VP3 were cotransfected into HEK293T cells for 48 h. Lysates were subjected to co-IP with anti-Flag mouse MAb and Western blotting with anti-Myc and anti-Flag rabbit pAbs. (F) Myc-VP3 or Myc-VP3ΔNLS was transfected into HEK293T cell for 24 h. The cytoplasmic elements (CE) and nuclear elements (NE) were extracted and subjected to immunoblotting analysis. GAPDH and histone 3 were used as markers of the cytosolic and nuclear fractions, respectively. (G) The distribution of VP3 in IBDV-infected DF-1 cells was analyzed by confocal assay. DF-1 cells were infected with IBDV for 12 h and then the cells were subjected to confocal assay with anti-VP3 mouse MAb. Nuclei were stained with DAPI. The top panel represents the white box of the bottom panel. (H) Relative intensity of the two channels along the white line in G. (I) Western blotting was used to detect the localization of VP3 in IBDV-infected DF-1 cells. DF-1 cells were infected with IBDV (MOI = 10) for the indicated time. The cytoplasmic elements (CE) and nuclear elements (NE) were extracted and subjected to immunoblotting analysis. (J) *In vitro*, VP3 binds API5 and SUMO3-API5. GST-VP3 was incubated with His-API5 or SUMO3-API5 for 4 h at 4°C. Then, GST resin was added. Finally, the pellets were lysed and subjected to Western blotting assays with the indicated antibodies. SUMO3-API5 was obtained as described for the *in vitro* SUMOylation assay. (K) VP3 deSUMOylates API5 *in vitro*. Api5-SUMO3 obtained in an *in vitro* SUMOylation reaction was incubated with purified recombinant His-VP3 expressed in E. coli BL21. The reaction products were analyzed by Western blotting with anti-API5 mouse MAb. (L) The CC3 domain of VP3 is required for SUMOylation inhibition of API5. HEK293T cells were cotransfected with Flag-API5, HA-SUMO3, and Myc-UBC9 together with wild-type Myc-VP3 or its truncation mutants Myc-VP3ΔCC1, Myc-VP3ΔCC2, and Myc-VP3ΔCC3 for 48 h. Cellular lysates were subjected to SUMOylation assays with anti-Flag mouse MAb and immunoblotting using anti-HA mouse MAb and anti-Flag and anti-Myc rabbit pAbs.

To explore if *Avibirnavirus* infection could interfere with the SUMOylation of API5, lysates from mock-infected and IBDV-infected DF-1 cells or chicken’s bursa of fabricius (BF, the IBDV-infected target organ) were subjected separately to SUMOylation assays. Figure 4A and B show that SUMOylated API5 in IBDV-infected cells and BF decreased markedly compared with that in mock-infected cells and BF. To further investigate whether VP3 was directly related to the *Avibirnavirus*-induced decrease of API5 SUMOylation, small interfering RNA (siRNA) against VP3 was used. Figure 4C shows that VP3 knockdown significantly inhibited IBDV-induced SUMO2/3-API5 reduction. Moreover, we observed that levels of SUMO3-API5 were reduced significantly in VP3-overexpressing HEK293T cells (Fig. 4D). These data clearly suggested that VP3 plays an important role for IBDV-induced reduction of API5 SUMOylation. Figure 1K and L demonstrate that SENP1 and SENP2 could reverse the SUMO3 modification of API5, while Fig. 4E shows that VP3 was not detected in SENP1- or SENP2- precipitations, indicating that the VP3-induced decrease in API5 SUMOylation is independent of SENP1 and SENP2.

Given that the SUMOylated API5 locates in the nucleus, we hypothesized that VP3 could translocate into the nucleus and deSUMOylate SUMO3-API5 by direct interaction. A potential nuclear localization signal (NLS) is predicted at residues 76 to 105 of the VP3 protein (R^76^AKYGTAGYGVEARGPTPEEAQRGKDTRIS^105^) by an online tool (NLS Mapper, http://nls-mapper.iab.keio.ac.jp/cgi-bin/NLS Mapper form.cgi). Subsequently, the cytoplasmic elements (CE) and nuclear elements (NE) of the cells transfected with wild-type Myc-VP3 or Myc-VP3ΔNLS were subjected to immunoblotting analysis. As shown in Fig. 4F, VP3, but not VP3ΔNLS, could be detected in the NE fraction. Moreover, VP3 could translocate into the nucleus during IBDV infection (Fig. 4G to I) and bind directly SUMO3-API5 in a GST pulldown assay (Fig. 4J). Furthermore, *in vitro* deSUMOylation assays demonstrated that the protein bands with high molecular weight detected in the lane corresponding to API5-SUMO3 were greatly reduced after incubation with VP3 (Fig. 4K). In cells, SUMOylation assays further confirmed that VP3 CC3, but not CC1 and CC2, was essential for decreasing SUMO3-conjugated API5 (Fig. 4L), suggesting that the VP3-mediated reduction of API5 SUMOylation depended on their interaction. Altogether, the viral protein VP3 CC3 domain might have an activity similar to deSUMOylation enzymes for directly inhibiting the SUMO3 modification of API5.

### VP3 inhibits API5 SUMOylation by facilitating UBC9 degradation.

**FIG 5:**
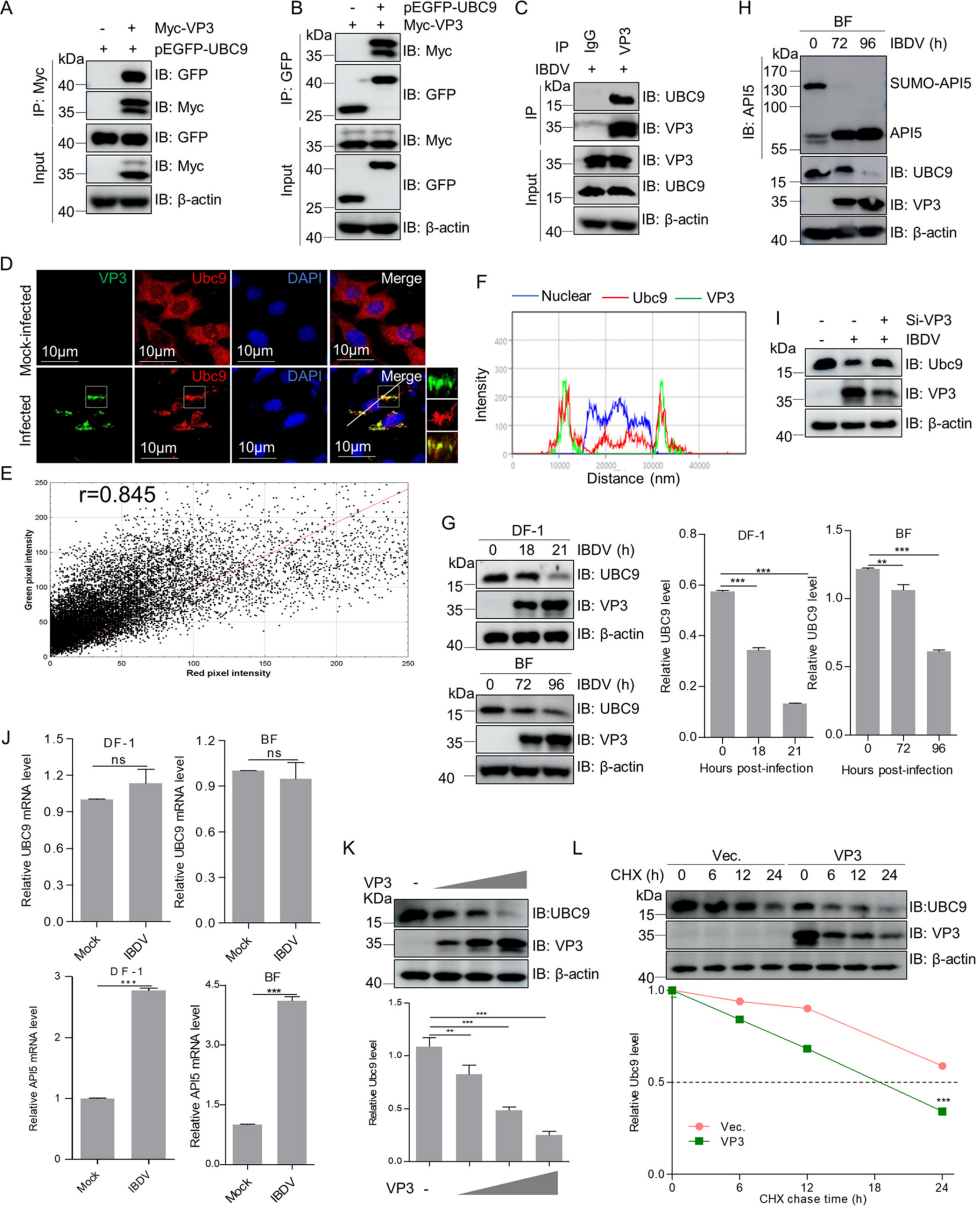
Viral protein VP3 inhibits API5 SUMOylation via promoting UBC9 degradation. (A and B) VP3 binds to UBC9 during transfection. Myc-VP3 and pEGFP-UBC9 were cotransfected into HEK293T cells for 48 h and lysates were subjected to a co-IP assay with anti-Myc or anti-GFP mouse MAb and to immunoblotting with anti-Myc and anti-GFP rabbit antibodies. (C) VP3 interacts with endogenous UBC9. DF-1 cells were infected for 12 h with IBDV. Lysates were subjected to a co-IP assay with anti-VP3 mouse MAbs and Western blotting with anti-VP3 and anti-UBC9 rabbit antibodies. (D) VP3 colocalizes with endogenous UBC9. DF-1 cells were infected with IBDV for 18 h and then incubated with anti-VP3 mouse MAb, anti-UBC9 rabbit pAb, FITC-labeled goat anti-mouse IgG, and Alexa Fluor 546-conjugated donkey anti-rabbit IgG. DAPI staining revealed the nuclei. The images were obtained by confocal microscopy. Scale bar, 10 μm. (E) Scatterplot. The Image J Just Another Colocalization Plugin (JACoP) was applied for quantitative colocalization analysis. In the scatterplot, the symbol *r* represents the Pearson's correlation coefficient. (F) Relative intensity of the three channels along the white line in D. (G and H) IBDV infection reduces API5 SUMOylation by decreasing UBC9 expression levels. Lysates from DF-1 cells infected with IBDV at an MOI of 1 and BF of 5-week-old specific-pathogen-free chickens infected with virulent IBDV strain NB at a dose of 0.1 ml (2 × 10^3^ 50% bird lethal doses/ml) for the indicated times were subjected to Western blotting with the indicated antibodies. (I) The IBDV-induced decrease of UBC9 protein is relevant to viral protein VP3. DF-1 cells were treated as described in Fig. 4C and then subjected to immunoblotting analysis with indicated antibodies. (J) The detection of the mRNA of *API5* and *UBC9* from IBDV-infected DF-1 cells and BFs. RNA extracted from IBDV-infected DF-1 cells or BF was subjected to qRT-PCR assays as described in the Materials and Methods. Data are the mean ± SD of three independent experiments. ***, *P < *0.001; ns, *P > *0.05; one-way ANOVAs. (K) VP3 promotes the degradation of UBC9 in a dose-dependent manner. PCDH-VP3 and empty vector were transfected separately into DF-1 cells at different doses for 36 h and the lysates were subjected to immunoblotting analysis with anti-UBC9 rabbit pAb. (L) VP3 shortens the half-life of UBC9. PCDH-VP3 and empty vector were transfected separately into DF-1 cells for 24 h and the resulting cells were treated with cycloheximide (CHX) (100 μg/ml, Medchemexpress, Monmouth Junction, NJ, USA), an inhibitor of protein synthesis, for the indicated times. The cellular lysates were used for Western blotting with anti-UBC9 pAb. The detection of β-actin served as a loading control.

To investigate if *Avibirnavirus* VP3 can bind to UBC9, co-IP assays were performed. Results showed that VP3 associated with UBC9 during transfection (Fig. 5A and B) and infection (Fig. 5C). Moreover, endogenous UBC9 in IBDV-infected cells colocalized with VP3 (Fig. 5D to F). In addition, immunoblotting results showed that the UBC9 protein level decreased markedly in comparison with that in mock-infected control cells and BF during virus infection (Fig. 5G). Meanwhile, we observed that the level of SUMOylated API5 decreased markedly, although IBDV infection upregulated the level of total API5 (Fig. 5H). These data indicated that IBDV infection might inhibit the SUMOylation of API5 by downregulating UBC9. To further investigate if the decrease of IBDV-induced UBC9 is relevant to VP3, the lysates of IBDV-infected DF-1 cells with siRNA against VP3 were detected by immunoblotting. Figure 5I shows that VP3 knockdown blocked the IBDV-induced UBC9 reduction, indicating that IBDV-induced decrease of UBC9 was viral protein VP3 dependent. However, in quantitative real-time reverse transcriptase PCR (qRT-PCR) assays, we observed no obvious changes in *UBC9* mRNA levels, although the *API5* mRNA level was upregulated markedly following IBDV infection (Fig. 5J). These findings revealed that IBDV infection facilitates the degradation of UBC9 protein. Figure 5A to F show the interaction and colocalization of VP3 and UBC9. Thus, to further explore whether VP3 was a direct regulator that accelerated UBC9 degradation, lysates of DF-1 cells transfected with expression plasmid encoding VP3 or empty vector were subjected to immunoblotting. Figure 5K and L show that VP3 overexpression enhanced UBC9 degradation in a dose-dependent manner, and shortened the half-life of UBC9. These data demonstrated that accelerating UBC9 degradation might be another mechanism by which VP3 decreases API5 SUMOylation.

### VP3 promotes UBC9 proteasome-dependent degradation by targeting ubiquitin E3 ligase TNF receptor-associated factor 3.

**FIG 6:**
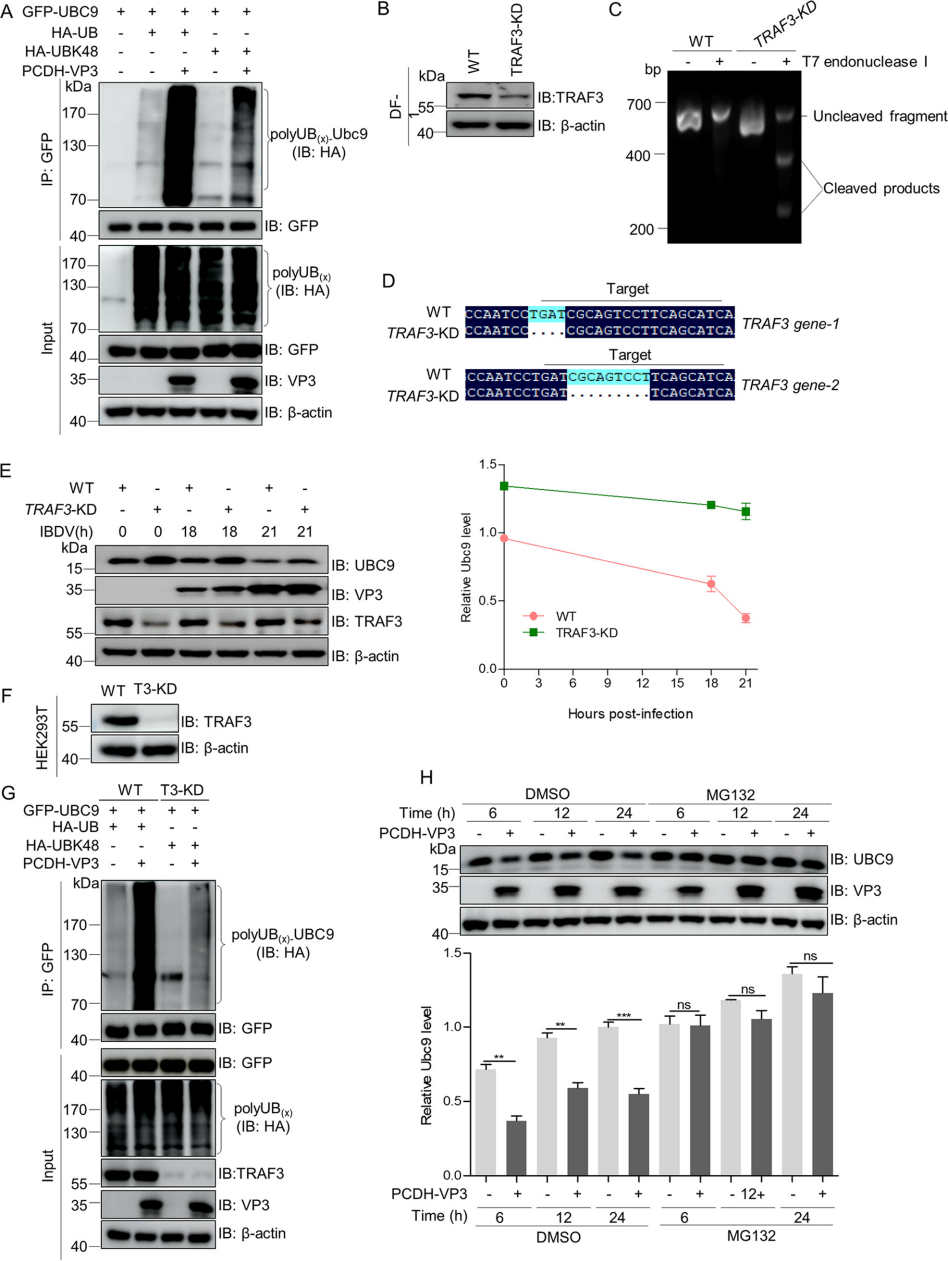
VP3 facilitates UBC9 proteasome-dependent degradation by recruiting TRAF3. (A) VP3 enhances K48-linked ubiquitination of UBC9. The lysates from HEK293T cells transfected with the indicated plasmids were subjected to ubiquitination assays using anti-GFP mouse MAb and immunoblotting with anti-GFP rabbit pAb and anti-HA and anti-VP3 mouse MAbs. (B to D) Generation of *TRAF3* knockdown (*TRAF3-*KD) DF-1 cells. Immunoblotting analysis of TRAF3 expression in WT and *TRAF3*-KD DF-1 cells (B). *TRAF3* fragment from WT and *TRAF3*-KD DF-1 cells was identified using T7 endonuclease I (EN303-01, Vazyme, Nanjing, China) according to the instructions (C). The primers for the amplification of the *TRAF3* fragment containing the Cas 9 target sequences: 5′-GTATGTATCACGTCCACATGATGTC-3′ and 5′-CAACAATTTTGACAATATTTACAG-3′. (D) *TRAF3* sequence comparison of WT and *TRAF3*-KD DF-1 cells. The *TRAF3* fragment with Cas 9 target sequence was cloned into pMD18-T vector. Multiple positive monoclonal colonies were sequenced. (E) TRAF3 is relevant to the degradation of UBC9 in DF-1 cells with IBDV. Wild-type and *TRAF3*-KD DF-1 cells were separately inoculated into 6-well plates and infected with IBDV at an MOI of 1 for the indicated time. The cellular lysates were subjected to Western blotting with anti-UBC9 rabbit pAb and anti-VP3 mouse MAb. (F) Construction of *TRAF3*-knockdown HEK293T cells (designated as T3-KD). The lysates from wild-type and T3-KD HEK293T cells were subjected separately to immunoblotting analysis for TRAF3 using anti-TRAF3 rabbit pAb. (G) VP3 increases ubiquitination of UBC9 by targeting the ubiquitin E3 ligase TRAF3. Wild-type and T3-KD HEK293T cells were cotransfected separately with GFP-UBC9 and HA-Ub or HA-UbK48 along with expression plasmid encoding VP3 or the corresponding empty vector. The cellular lysates were then subjected to ubiquitination assays with anti-GFP mouse MAb and immunoblotting analysis with anti-GFP rabbit pAb, anti-HA, and anti-VP3 mouse MAbs. (H) The ubiquitin proteasome-dependent degradation system is essential for VP3 to accelerate the degradation of endogenous UBC9. DF-1 cells were transfected with PCDH-VP3 or the corresponding empty vector for 24 h and the resulting cells were treated with MG132 (10 μM, Beyotime) to block proteasome-mediated protein degradation for the indicated time. Dimethyl sulfoxide (DMSO) served as the negative control. The cellular lysates were subjected to Western blotting with the indicated antibodies. The β-actin expression served as a loading control.

K48-linked ubiquitination of substrate proteins commonly regulates protein stability (46). To explore the detailed molecular mechanism by which VP3 targets UBC9 for degradation, ubiquitination assays of UBC9 were carried out in HEK293T cells transfected with the indicated plasmids. We observed that VP3 overexpression greatly enhanced K48-linked ubiquitination of UBC9 (Fig. 6A), suggesting that VP3 might facilitate UBC9 degradation via the ubiquitin proteasomal system.

Tumor necrosis factor (TNF) receptor-associated factor 3 (TRAF3) is an important regulator of nuclear transcription factor-kappa B (NF-κB) and type I interferon activation (47), and it has been reported that the severe acute respiratory syndrome coronavirus (SARS-CoV) protein ORF3a activates the NLRP3 inflammasome by promoting TRAF3-mediated caspase recruitment domain ubiquitination (48). To confirm whether UBC9 degradation driven by IBDV was related to TRAF3, *TRAF3* knockdown DF-1 cells (*TRAF3*-KD) were constructed using Cas9 enzyme (Fig. 6B to D) and infected with IBDV. Immunoblotting results showed that IBDV-induced UBC9 degradation was decreased in *TRAF3*-KD DF-1 cells compared with that in control cells (Fig. 6E). Moreover, in cells, SUMOylation assays showed that in comparison with control HEK293T cells, TRAF3 knockdown not only markedly decreased the ubiquitination of UBC9 but also inhibited the increase of VP3-mediated UBC9 ubiquitination (Fig. 6F and G), suggesting that TRAF3 was required for VP3 to promote UBC9 proteasomal degradation. To further confirm that the VP3-induced UBC9 degradation was proteasome dependent, we assessed the effect of the proteasome inhibitor MG132 on VP3-mediated UBC9 degradation in DF-1 cells. Figure 6H shows that MG132 treatment inhibited VP3-mediated degradation of UBC9. Collectively, these findings revealed that TRAF3 acts as a ubiquitin E3 ligase of UBC9 ubiquitination and that VP3 facilitates UBC9 proteasome-dependent degradation by recruiting TRAF3.

### Inhibition of API5 on IBDV replication depends on its K404 SUMOylation.

**FIG 7:**
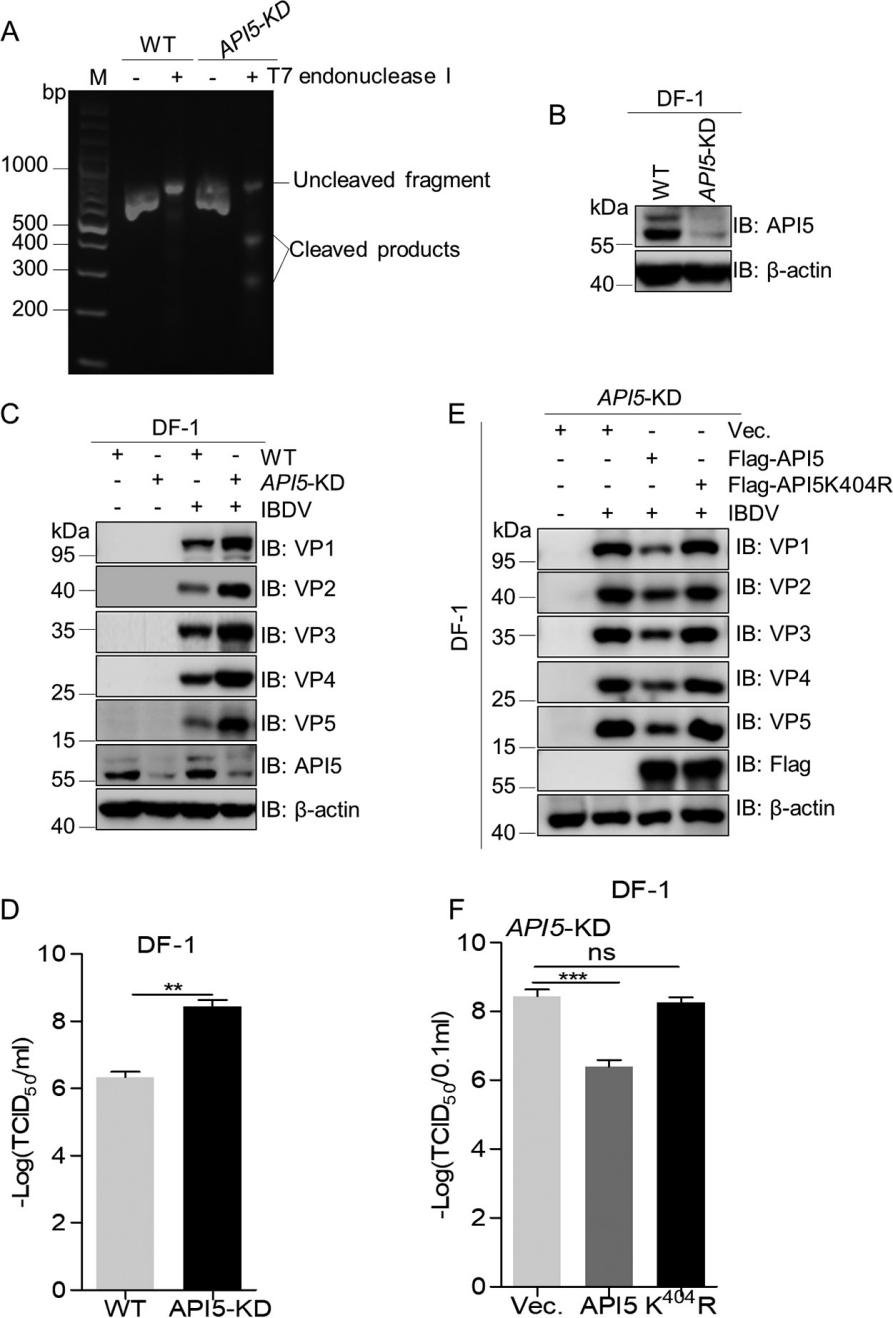
Impacts of API5-WT and API5-K404R on IBDV replication. (A and B) Construction of API5 knockdown (*API5*-KD) DF-1 cells. The *API5* genes and cellular lysates from WT or *API5*-KD DF-1 cells were separately subjected to T7 endonuclease I and immunoblotting assays. The primers used for the amplification of the *API5* fragment with Cas 9 target sequence were 5′-GAACAGGTCCTGGAACCAAAAG-3′ and 5′-CAGAGCTATTCTGTTGATCAATG-3′. (C) WT and *API5*-KD DF-1 cells were infected with IBDV (MOI = 0.1) or mock infected for 24 h, after which the lysates were immunoblotted with the indicated viral protein antibodies. (D) Detection of virus titer in C. (E and F) *API5*-KD DF-1 cells were transfected with WT and K404R mutant API5 or corresponding empty vector for 24 h using JetPRIME transfection reagent and were then infected with IBDV at an MOI of 0.1 for 24 h. Expression levels of viral proteins and viral titers were assessed by immunoblotting (E) using the indicated viral protein antibodies and TCID_50_ (F) assays, respectively. The β-actin expression served as a loading control.

Previous study demonstrated that replication of influenza A virus can be suppressed by API5 (30). To evaluate the impacts of API5 on IBDV replication, *API5* knockdown DF-1 cells (*API5*-KD) were generated (Fig. 7A and B). The levels of viral protein and virus titers in WT and *API5*-KD cells with IBDV were determined by immunoblotting and 50% tissue culture infectious dose (TCID_50_) assays, respectively. As shown in Fig. 7C and D, virus titer and viral protein level were increased in IBDV-infected *API5*-KD DF-1 cells compared with that in WT cells, indicating that API5 plays a role as a negative regulator in IBDV replication. Subsequently, to investigate if the inhibition of API5 on IBDV proliferation depended on SUMO3 modification of API5 at the K404 site, *API5*-KD DF-1 cells restored with API5-WT, API5-K404R, or empty vector were infected with IBDV. Results showed that levels of viral protein and virus titer were greatly decreased in *API5*-KD DF-1 cells restored with API5-WT compared with that in cells containing mutant API5-K404R or empty vector (Fig. 7E and F), indicating that API5 K404 SUMO3 modification is essential for resisting IBDV infection. Altogether, these data indicated that the de-SUMOylation of API5 at K404 plays an important role in supporting IBDV replication.

### Residue K404 SUMOylation of API5 is critical for MDA5-dependent IFN-β induction.

**FIG 8:**
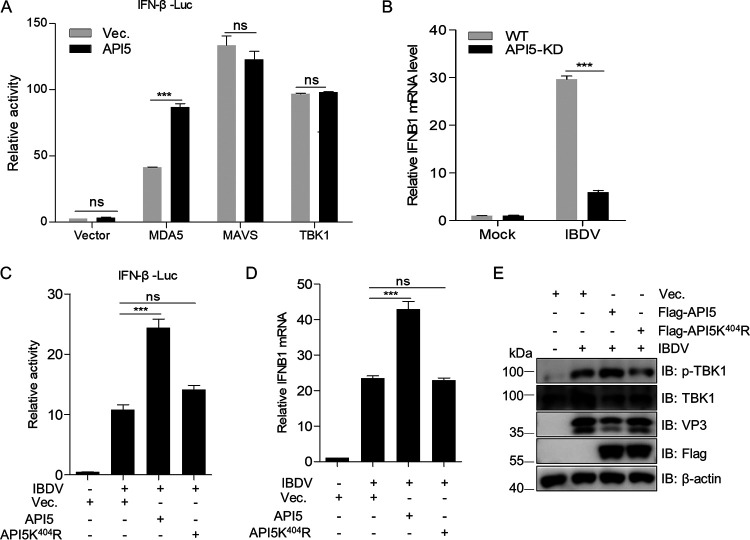
SUMOylated API5 promotes MDA5-dependent IFN-β induction. (A) API5 significantly enhances MDA5-mediated activation of the IFN-β promoter. HEK293T cells were transfected with the IFN-β reporter plasmid pRL-TK or empty vector, MDA5, MAVS, or TBK1 along with Flag-API5 or corresponding empty vector for 36 h, after which the IFN-β activity was measured with the dual-luciferase assay. (B) API5 knockdown represses the expression of *IFNB1* mRNA. Control and *API5* knockdown DF-1cells (*API5*-KD) were separately infected with IBDV (MOI = 10) for 12 h. The levels of *IFNB1* and *GAPDH* mRNA were detected by qRT-PCR. (C) SUMOylated API5 increases the activation of the IFN-β promoter induced by IBDV. *API5*-KD DF-1 cells were cotransfected with IFN-β reporter plasmid pRL-TK and wild-type API5, mutant API5K^404^R, or empty vector for 36 h using JetPRIME transfection reagent, after which the cells were infected with IBDV (MOI = 10) for 12 h. The cellular lysates were assessed by dual luciferase assay. (D and E) SUMOylated API5 enhances the transcription of the *IFNB1* gene and activation of TBK1. *API5*-KD DF-1 cells were transfected with wild-type API5, mutant API5K^404^R, or empty vector for 36 h, following by infection with IBDV for 12 h. The cells were harvested and subjected to qRT-PCR (D) and Western blotting (E) detection. The cells used for the detection of phosphorylation indexes were lysed in reporter gene cell lysate (RG027-1, Beyotime).

Beta interferon (IFN-β) was reported to play a critical role in inhibiting viral replication (49). IBDV dsRNA could be sensed by MDA5 (cytoplasmic RIG-I-like receptor melanoma differentiation gene 5) to induce IFN‐β expression (44, 50, 51). Therefore, we speculated that API5 might play an important role in regulating MDA5-induced IFN-β production. As shown in Fig. 8A, API5 significantly enhanced IFN-β activation triggered by MDA5 instead of MAVS (mitochondrial antiviral signaling protein) or TBK1 (TANK-binding kinase 1). Consistent with this, the mRNA transcript of *IFNB1* was downregulated in IBDV-infected *API5* knockdown cells compared with IBDV-infected wild-type cells (Fig. 8B), demonstrating that API5 significantly activated the MDA5-mediated IFN-β signaling pathway. Additionally, given that SUMOylation of substrates is involved in antiviral innate immunity (52–54) and API5 is a SUMOylated protein (Fig. 1), we speculated that the role of API5 on IBDV-driven IFN-β production might be relevant to its SUMOylation. As shown in Fig. 8C to E, in IBDV-infected *API5* knockdown cells, the activity of IFN-β promoter and the levels of *IFNB1* mRNA and phosphorylated TBK1 were significantly enhanced by restoring wild-type API5, but not API5K404R, suggesting that the SUMOylation of residue K404 of API5 is critical for upregulation of IFN-β expression. Overall, these results demonstrated that SUMOylation of API5 K404 contributed to IBDV-driven MDA5-dependent IFN-β production.

## DISCUSSION

SUMOylation has been reported to have an important role in modulating protein function under conditions of physical or other types of stress (55). Growing evidence reveals that SUMOylation of substrates affects their subcellular distribution and activity (56). In addition, nuclear targeting proteins prefer to be attached to SUMO moieties (57, 58). Antiapoptotic protein API5 was initially identified as a nuclear protein that protects cells from apoptosis and, although its lysine 251 acetylation is related to this antiapoptotic function, it was observed to be irrelevant to API5’s subcellular localization (20, 59, 60). However, to date, SUMOylation of API5 has not been reported. In this study, we first identified API5 as a SUMOylated protein that is localized mainly in the nucleus and showed that the K404 residue can be conjugated to SUMO3. Interestingly, a SUMO3 modification-deficient API5 mutant failed to locate to the nucleus, implying that SUMO3 modification at K404 contributes to API5 nuclear localization. Similarly, phosphatase and tensin homolog and extracellular-signal-regulated kinase 5 proteins could be efficiently modified by SUMO, contributing to their nuclear localization, and their SUMOylation-deficient mutant variants were not retained in nuclei (61–63).

Previous studies have shown that viral proteins can interfere with the SUMO system by different mechanisms, including interacting with SUMO pathway proteins (15), mimicking SUMO E3 ligases (64–66), inhibiting SUMO modification of substrate proteins (67), targeting UBC9 (16), and exhibiting SUMO protease activity (68). Our findings demonstrated that *Avibirnavirus* infection reduces the SUMO3-conjugation of API5 by facilitating degradation of UBC9. In parallel to its deSUMOylation, *Avibirnavirus* infection induces API5 to colocalize with the viral protein VP3 in the cytoplasm. Further investigation of the molecular mechanism indicated that viral protein VP3 directly inhibits the SUMOylation of API5 by targeting API5 and promoting UBC9 proteasome-dependent degradation through binding to the ubiquitin E3 ligase TRAF3. Thus, we propose that *Avibirnavirus* VP3 protein has a similar SUMO protease activity. As to whether viral protein VP3 is a new member of the SUMO-specific protease family, further investigation is required.

SUMOylation is a vital regulator of antiviral innate immunity (69, 70). Increasing evidence demonstrates that many host proteins involved innate immunity can be SUMOylated (71). During infection, viruses ensure their replication and infection through inhibition or induction of SUMOylation of cellular proteins implicated in antiviral defense. Ebola Zaire virus VP35 protein increases SUMOylation of interferon regulatory factor 7 and represses type I IFN transcription, leading to the enhancement of virus infection (52). Vesicular stomatitis virus infection reduces SUMOylation of DEAD/H (Asp-Glu-Ala-Asp/His) box polypeptide 39A (DDX39A) to repress nuclear export of antiviral transcripts for limiting IFN-β production and promoting viral replication (53). In this study, we revealed that SUMOylated API5 played a role as a positive regulator in antiviral innate immunity, and that IBDV VP3 deSUMOylated API5 to block MDA5-dependent IFN-β production and thereby support viral proliferation (Fig. 4, Fig. 8). This is the first reveal of the mechanism by which IBDV VP3 negatively regulates MDA5-dependent antiviral innate immunity by API5 deSUMOylation.

Additionally, our experiments demonstrated that SUMOylated API5 mainly localizes in the nucleus (Fig. 2B and C) and the deSUMOylation of API5 by viral protein VP3 caused translocation to the cytoplasm (Fig. 3A). Further, IBDV infection upregulated the expression of *API5* mRNA (Fig. 5J). *Avibirnavirus* VP3 protein has been reported to inhibit antiviral innate immunity by blocking viral double-stranded RNA binding to MDA5 (44), and API5 is a RNA-binding protein and promotes mRNA nuclear export (72). Considering that deSUMOylated API5 promotes IBDV replication, it needs further investigation as to whether the deSUMOylated API5 blocks the viral dsRNA binding to MDA5 for inhibiting IFN-β induction.

**FIG 9:**
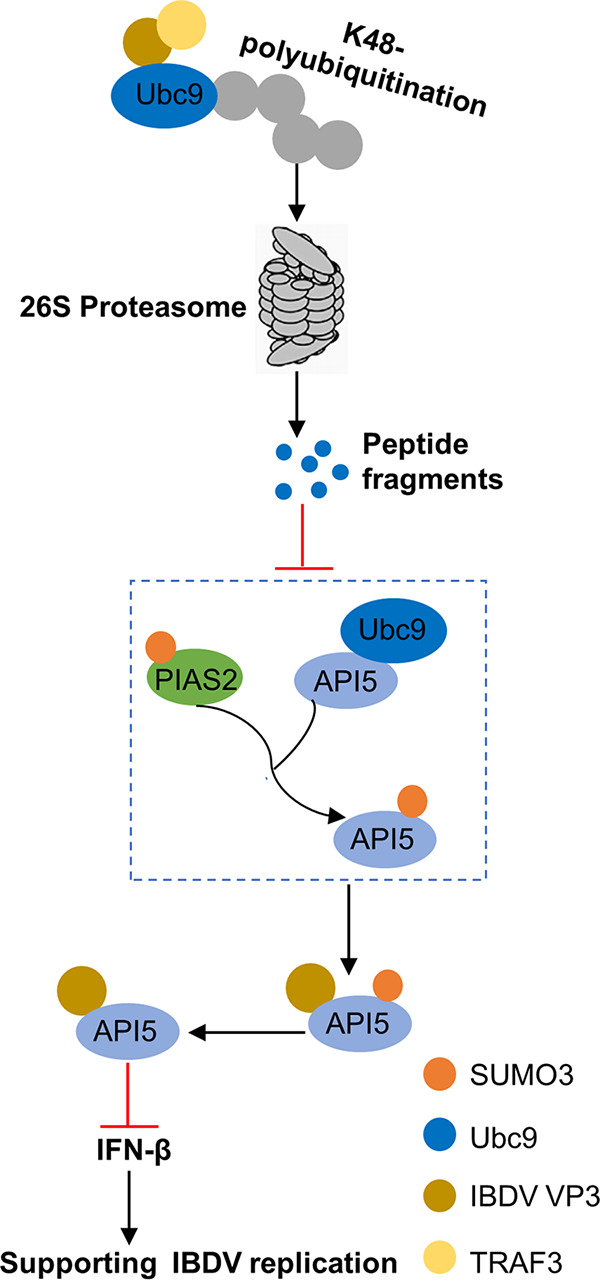
A proposed model for the inhibition of API5 SUMOylation mediated by IBDV VP3 protein. IBDV VP3 protein decreases API5 SUMOylation by targeting SUMOylated API5 and facilitating UBC9 proteasome-dependent degradation via targeting TRAF3, leading to the suppression of IFN-β induction for the enhancement of IBDV replication. The blue box represents the process of API5 SUMO conjugation mediated by PIAS2, E3 SUMO ligase.

In summary, we first identified that API5 is a SUMOylated protein and that Lys404 is an important SUMO3 modification site. In addition, we showed that IBDV infection decreases the SUMOylation of API5 and that viral protein VP3 directly inhibits the SUMOylation of API5 to block IFN-β induction for supporting virus replication by targeting API5 and promoting UBC9 proteasome-dependent degradation via binding to TRAF3 (Fig. 9). Taken together, using *Avibirnavirus* IBDV as a model of a nonenveloped RNA virus, we revealed the fine-tuned regulation mechanism of the conversion between SUMOylation and deSUMOylation of API5 upon virus infection and provided new ideas for the design of anti-IBDV agents.

## MATERIALS AND METHODS

### Cells and virus.

DF-1 cells (chicken embryo fibroblast; American Type Culture Collection [ATCC, Manassas, VA, USA] CRL-12203) and HEK293T cells (human embryonic kidney; ATCC CRL-11268) were cultured in Dulbecco’s modified Eagle’s medium (DMEM, Gibco, Carlsbad, CA, USA) supplemented 10% fetal bovine serum (FBS) and 1% penicillin-streptomycin. IBDV strain NB in our laboratory has been described previously (73, 74).

### Reagents and antibodies.

Anti-Flag (F1804), anti-Myc (M5546), and anti-HA (H3663) mouse monoclonal antibodies (MAbs) for immunoprecipitation (IP) were purchased from Sigma-Aldrich (St. Louis, MO, USA). Anti-Myc (R1208-1) and anti-Flag (0912-1) rabbit polyclonal antibodies (pAbs), anti-green fluorescent protein (GFP) rabbit MAb (ET1602-7) and anti-GST (EM80701), anti-β-actin (EM21002), and anti-histone 3 (M1306-4) mouse MAbs for Western blotting were purchased from HuaAn Biotechnology (Hangzhou, China). Anti-GAPDH (glyceraldehyde-3-phosphate dehydrogenase) rabbit pAb (AB-P-R001) was purchased from GoodHere Technology (Hangzhou, China). Anti-GFP (sc-9996) mouse MAb for IP was purchased from Santa Cruz Biotechnology (Santa Cruz, CA, USA). Anti-TRAF3 (D260776-0100) rabbit pAb was purchased from Sangon Biotech (Shanghai, China). Phospho-TBK1 (no. 5483) rabbit MAb was purchased from Cell Signaling Technology (Danvers, MA, USA). Anti-TBK1 (A2573) rabbit pAb was made by ABclonal (Wuhan, China). Dual luciferase reporter gene assay kit (RG027) was purchased from Beyotime (Shanghai, China). Mouse MAbs against IBDV viral proteins and anti-UBC9 rabbit pAb were prepared in our laboratory (75, 76). Anti-SUMO2/3 and anti-API5 rabbit pAbs and anti-API5 mouse MAb were produced in our laboratory (unpublished data). Dithiothreitol (DTT) (D8220-1g), ATP (A9130-1g), and phenylmethanesulfonyl fluoride (PMSF) (P8340) were purchased from Solarbio (Shanghai, China). The ExFect (T101-01/02) and JetPRIME (PT-114-15) transfection reagents were purchased from Vazyme Biotechnology (Nanjing, China) and Polyplus (New York, NY, USA), respectively.

### Constructs.

Gallus *API5* ORF, amplified from DF-1 cells, was separately subcloned into vectors p3×FLAG-CMV-10 (Clontech, Palo Alto, CA, USA) and pET-28a (Novagen, Madison, WI, USA). All Flag-API5 mutants were obtained by site-specific mutagenesis. *PIAS1* and *PIAS2* fragments obtained from DF-1 cells and the *SENP2* ORF amplified from HEK293T cells were cloned separately into vector pCMV-Myc-N (635689; Clontech). Myc-SENP2cs were constructed by substitution mutation. The *TRAF3* fragment obtained from DF-1 cells was inserted into vector pCMV-Flag-N (635688; Clontech). Chicken expression plasmids for Flag-tagged MDA5, MAVS, and TBK1 had been stored in our laboratory. Chicken IFN-β promoter luciferase reporter plasmid, Myc-VP3 and its truncated mutants Myc-VP3ΔCC1, Myc-VP3ΔCC2 and Myc-VP3ΔCC3, pET-28a-VP3, pGEX-4T-1-VP3, shUBC9#1, shUBC9#2, shCON, Myc-SENP1, Myc-SENP1_C603S_, Flag-SENP1, Flag-SENP2, HA-SUMO3, Myc-Ubc9, pEGFP-Ubc9, HA-SUMO3_G92A_, Myc-Ubc9_C93S_, HA-Ub, HA-UbK48, pET-28a-SUMO3, pET-28a-Ubc9, pET-28a-SAE1, and pET-28a-SAE2 have been described previously (35, 43, 44, 77). The primers used are listed in Table 1.

**TABLE 1:** The primers used for constructs

Name	Forward sequence (5′–3′)	Reverse sequence (5′–3′)
*API5-*K36R	CTTGGATGGCGTGAGAGGAGGCGCCAAGG	CCTTGGCGCCTCCTCTCACGCCATCCAAG
*API5*-K126R	CTTTGCTCAGTATATTTAGGATGGATGCTAAAG	CTTTAGCATCCATCCTAAATATACTGAGCAAAG
*API5*-K404R	CAGGAGAAGCCTTGAGAACAGAAGAGAACAAG	CTTGTTCTCTTCTGTTCTCAAGGCTTCTCCTG
*API5-*K474R	GCTTCGGCAGGACCAAGAAGGGATGCCAGGC	GCCTGGCATCCCTTCTTGGTCCTGCCGAAGC
*SENP2*-C548S	CTGAATGGGAGTGATTCGGGAATGTTTACTTG	CAAGTAAACATTCCGAATCACTCCCATTCAG
*VP3-*Q205A	CGTGGCCCAAACGCAGAACAGATGAAAGATC	GATCTTTCATCTGTTCTGCGTTTGGGCCACG
*VP3-*E206A	CGTGGCCCAAACCAAGCACAGATGAAAGATCTG	CAGATCTTTCATCTGTGCTTGGTTTGGGCCACG
*VP3-*Q207A	GGCCCAAACCAAGAAGCAATGAAAGATCTGCTC	GAGCAGATCTTTCATTGCTTCTTGGTTTGGGCC
*VP3-*M208A	CCAAACCAAGAACAGGCAAAAGATCTGCTCTTG	CAAGAGCAGATCTTTTGCCTGTTCTTGGTTTGG
*VP3-*K209A	CAAACCAAGAACAGATGGCAGATCTGCTCTTGACTG	CAGTCAAGAGCAGATCTGCCATCTGTTCTTGGTTTG
*VP3-*D210A	CAAGAACAGATGAAAGCACTGCTCTTGACTGCG	CGCAGTCAAGAGCAGTGCTTTCATCTGTTCTTG
*VP3-*L211A	GAACAGATGAAAGATGCACTCTTGACTGCGATG	CATCGCAGTCAAGAGTGCATCTTTCATCTGTTC
*VP3-*L212A	CAGATGAAAGATCTGGCATTGACTGCGATGGAG	CTCCATCGCAGTCAATGCCAGATCTTTCATCTG
*VP3-*L213A	ATGAAAGATCTGCTCGCAACTGCGATGGAGATG	CATCTCCATCGCAGTTGCGAGCAGATCTTTCAT
*VP3-*T214A	GAAAGATCTGCTCTTGGCAGCGATGGAGATGAAG	CTTCATCTCCATCGCTGCCAAGAGCAGATCTTTC
*VP3-*M216A	CTGCTCTTGACTGCGGCAGAGATGAAGCATCGC	GCGATGCTTCATCTCTGCCGCAGTCAAGAGCAG
*VP3-*E217A	CTCTTGACTGCGATGGCAATGAAGCATCGCAATC	GATTGCGATGCTTCATTGCCATCGCAGTCAAGAG
*VP3-*M218A	GACTGCGATGGAGGCAAAGCATCGCAATC	GATTGCGATGCTTTGCCTCCATCGCAGTC
*VP3-*K219A	GACTGCGATGGAGATGGCACATCGCAATCCCAGG	CCTGGGATTGCGATGTGCCATCTCCATCGCAGTC
*VP3*ΔNLS	GGCAGCAAGTCGCAAAAGAAGATGGAGACC	GGTCTCCATCTTCTTTTGCGACTTGCTGCC

### Expression and purification of recombinant proteins.

Escherichia coli BL21 (pLysS) cells harboring pET-28a-API5, pET-28a-SUMO3, pET-28a-UBC9, pET-28a-SAE1, pET-28a-SAE2, pET-28a-VP3, or pGEX-4T-1-VP3 plasmid were cultured separately in 200 ml of Luria-Bertani (LB) medium and induced with 1 mM isopropyl β-D-thiogalactopyranoside (IPTG) at 16°C overnight. Cell pellets were lysed by sonication in binding buffer with 1 mM PMSF (50 mM Tris-HCl, 10 mM imidazole [pH 8.0] for His-tagged proteins; 50 mM Tris-HCl, 150 mM NaCl [pH 8.0] for GST-VP3). After centrifugation, the supernatant was incubated separately with Ni-NAT and GST resin for 2 h at 4°C and then purified. His-tagged proteins and GST-VP3 protein were eluted in eluting buffer containing 80 mM imidazole and containing 2 mg/ml of reduced glutathione, respectively.

### Western blotting, immunofluorescence (IF) staining, cellular fractionation, and dual luciferase assay.

These processes were previously described (51).

### Co-IP and GST pulldown assays.

For co-IP, cells were lysed in NP-40 buffer (P0013F; Beyotime, Shanghai, China) containing 1 mM PMSF for 4 h or overnight at 4°C. After centrifugation, the supernatant was incubated with the indicated antibodies and protein A/G PLUS-agarose at 4°C for 4 h or overnight. For the GST pulldown assay, purified GST and GST-VP3 protein were incubated separately with His-API5 at 4°C for 4 h and the GST resin was added. After centrifugation, the pellets were washed five times with NP-40 buffer at 4°C, and then lysed for immunoblotting analysis.

### SUMOylation assay.

For SUMOylation in cells, HEK293T cells cotransfected with the indicated plasmids, DF-1 cells, and chicken bursa of Fabricius with or without IBDV, were lysed in NP-40 buffer or radioimmunoprecipitation assay (RIPA) buffer (50 mM tris-base, 150 mM NaCl, 1% Triton X-100, 1% sodium deoxycholate and 1% SDS [pH 7.4 to 7.6]) containing PMSF and *N*-ethylmaleimide (NEM, E3876; Sigma-Aldrich), a deSUMOylase inhibitor. The supernatant was subjected to co-IP assays.

The *in vitro* SUMOylation assay was performed as described previously (77–79). Briefly, 50 μl of SUMOylation buffer (50 mM Tris-HCl [pH 7.5], 50 mM KCl, 5 mM MgCl, 1 mM ATP, 1 mM DTT) was mixed with 2 μg of substrate protein Api5, 0.5 μg of SAE1/SAE2, 2 μg of UBC9, 5 μg of SUMO3, 2 U of creatine phosphokinase (Merck, Darmstadt, Germany), 3 U of inorganic pyrophosphatase (Merck), and 5 μl of 100 mM creatine phosphate sodium salt (Macklin, Shanghai, China). The mixture was incubated at 37°C for 1 to 1.5 h. Finally, the reaction products were fractionated on 8% SDS-PAGE and analyzed by Western blotting with anti-API5 mouse MAb.

### *In vitro* deSUMOylation.

API5-SUMO3 obtained in an *in vitro* SUMOylation reaction was incubated with recombinant VP3 in reaction buffer (50 mM Tris [pH 7.5], 2 mM MgCl_2_, and 5 mM β-mercaptoethanol) at 37°C for 1 h. Finally, the reaction was stopped by the addition of 20 μl of SDS sample buffer containing β-mercaptoethanol. The prepared samples were subjected to SDS-PAGE and immunoblotting.

### Ubiquitination assay.

HEK293T cells transfected with the indicated plasmids were treated with MG132 (S1748-5mg; Beyotime) (10 μM) for 12 h before harvesting the samples. The supernatant of the cellular lysates was subjected to co-IP assays.

### Construction of *API5*-KD and *TRAF3*-KD cells.

The chicken *API5* (*chAPI5*) gene target sequence (5′-GCCTACCAGGTGATCTTGGA-3′), the chicken *TRAF3* (*chTRAF3*) gene target sequence (5′-GATGCTGAAGGACTGCGATC-3′), and the human *TRAF3* (*hTRAF3*) gene target sequence (5′-AGCCCGAAGCAGACCGAGTG-3′) were separately inserted into the guide RNA (gRNA) expression plasmid, PX 459 (Addgene, Watertown, MA, USA). HEK293T and DF-1 cells were transfected separately with the indicated recombinant constructs for 36 h and then were selected using 10 μg/ml puromycin (58-58-2; InvivoGene, San Diego, CA, USA) for another 36 h. The resulting DF-1 cells were inoculated into 96-well plates for colony formation of monoclonal cells by the limited dilution method. Each colony was separately transferred into 24-well plates. The transfected HEK293T cells were not subcloned.

### Virus titer detection.

The harvested cells were freeze-thawed three times and, after centrifugation (12 000 × *g*), the supernatants were subjected to virus titer detection as described previously (35).

### Quantitative real-time reverse transcription PCR (qRT-PCR).

Total RNA was isolated from DF-1 cells or BF using the TRIzol reagent (Invitrogen) according to the manufacturer’s instructions. Then, 1 μg RNA was reverse transcribed into cDNA using the PrimeScript RT reagent kit with gDNA Eraser (RR047A; TaKaRa, Kusatsu, Japan) according to the manufacturer’s instructions. The mRNAs of indicated genes were amplified using TB Green Premix *Ex Taq* (Tli RNaseH Plus) (RR420A; TaKaRa). *GAPDH* was amplified to normalize the relative abundance of the indicated mRNAs. The primers for qRT-PCR are listed in Table 2.

**TABLE 2:** Quantitative real-time PCR primers

Gene	Forward sequence (5′–3′)	Reverse sequence (5′–3′)
*API5*	ACAACCTCAAGTTCACCTCCAA	ACTACGATTGCCACGTCCTC
*UBC9*	AAGAAGGGGACACCATGGGA	TGGGGGTGAAGAAGGGTAGT
*GAPDH*	CCAGCAACATCAAATGGGCAGAT	TGATAACACGCTTAGCACCACCCT
*IFNB1*	ACCAGGATGCCAACTTCTCTTGGA	ATGGCTGCTTGCTTCTTGTCCTTG

### Statistical analysis.

Statistical differences were assessed using one-way analysis of variance (ANOVA) using SPSS Statistics 20 (IBM Corp., Armonk NY, USA). The results of all statistical analysis are shown as the mean ± SD (standard deviation) of at least two independent experiments. For all experiments, *P < *0.05 was considered statistically significant. *, *P < *0.05; **, *P < *0.01; ***, *P < *0.001; ns, *P > *0.05.
